# Supplementary material for: Risk factors for neuropsychiatric symptoms in patients with Parkinson’s disease during COVID-19 pandemic in Japan
Source: PLoS One. 2021 Jan 22;16(1):e0245864. doi: 10.1371/journal.pone.0245864 (PMC7822544; doi:10.1371/journal.pone.0245864)
Supplement: S1 Table — (DOCX) [file pone.0245864.s001.docx]

| **Table S1. Demographic Characteristics of Responders stratified by sex** | | | | | |
| --- | --- | --- | --- | --- | --- |
|  |  | **No. (%)** |  |  |  |
| **Characteristics** | | **Total** | **female** | **male** | **P-value** |
| Disease duration, years | |  |  |  |  |
|  | < 5 | 22 (56.4) | 7 (50.0) | 15 (60.0) | 0.54 |
|  | ≥ 5 | 17 (43.5) | 7 (50.0) | 10 (40.0) |  |
| HY stage | |  |  |  |  |
|  | stage 0-2 | 12 (30.7) | 4 (28.5) | 8 (32.0%) | 0.82 |
|  | stage 3, 4 | 27 (69.2) | 10 (71.5) | 17 (68.0%) |  |
| Non-motor symptoms | |  |  |  |  |
|  | Cognitive impairment | 6 (15.3) | 3 (21.4) | 3 (12.0) | 0.43 |
|  | Hallucinations | 3 (7.6) | 1 (7.1) | 2 (8.0) | 0.92 |
| RBD | | 6 (15.3) | 1 (7.1) | 5 (20.0) | 0.28 |
| L-DOPA, mg | |  |  |  |  |
|  | < 600 | 30 (76.9) | 10 (71.4) | 20 (80.0) | 0.54 |
|  | ≥ 600 | 9 (23.0) | 4 (28.5) | 5 (20.0) |  |
| Other medications | |  |  |  |  |
|  | Dopamine agonist | 15 (38.4) | 7 (50.0) | 8 (80.0) | 0.26 |
|  | Psychiatric medicines | 8 (20.5) | 3 (21.4) | 5 (20.0) | 0.91 |
|  | Sleeping medicines | 13 (33.3) | 4 (28.5) | 9 (36.0) | 0.63 |
| MDS-UPDRS part 2, | | 17 (10-20.5) | 18.5 (16-24) | 16 (10-19.25) | 0.31 |
| median (IQR), n=36* | |  |  |  |  |
| Abbreviations: HY, Hoehn & Yahr; RBD, REM sleep behavior disorder; MDS-UPDRS, Movement Disorder Society Unified Parkinson's Disease Rating Scale; IQR, interquartile range. | | | | | |
| *Of the 39 participants, 36 responded to the question about MDS-UPDRS part 2. | | | | | |
