# Supplementary material for: Risk factors for neuropsychiatric symptoms in patients with Parkinson’s disease during COVID-19 pandemic in Japan
Source: PLoS One. 2021 Jan 22;16(1):e0245864. doi: 10.1371/journal.pone.0245864 (PMC7822544; doi:10.1371/journal.pone.0245864)
Supplement: S2 Table — a. Severity categories of depression, anxiety, and insomnia measurements in female PD patients and controls. b. Severity categories of depression, anxiety, and insomnia measurements in male PD patients and controls. (DOCX) [file pone.0245864.s002.docx]

| **Table S2a. Severity Categories of Depression, Anxiety, and Insomnia Measurements in Female PD Patients and Controls** | | | | | |
| --- | --- | --- | --- | --- | --- |
| **[Female]** | | **No. (%)** |  |  |  |
| **Severity category** | | **Total** | **PD** | **Control** | **P-value*** |
| PHQ-9, depression symptoms | | n = 40 | n = 14 | n = 26 |  |
|  | Normal | 20 (50.0) | 6 (42.8) | 14 (53.8) | **0.001** |
|  | Mild | 12 (30.0) | 1 (7.1) | 11 (42.3) |  |
|  | Moderate | 3 (7.5) | 2 (13.2) | 1 (3.8) |  |
|  | Severe | 5 (12.5) | 5 (35.7) | 0 (0) |  |
| GAD-7, anxiety symptoms | | n = 41 | n = 14 | n = 27 |  |
|  | Normal | 20 (48.7) | 7 (50.0) | 13 (48.1) | 0.424 |
|  | Mild | 13 (31.7) | 2 (14.2) | 11 (40.7) |  |
|  | Moderate | 4 (9.7) | 1 (7.1) | 3 (11.1) |  |
|  | Severe | 4 (9.7) | 4 (28.5) | 0 (0) |  |
| ISI, insomnia symptoms | | n = 41 | n = 14 | n = 27 |  |
|  | Absence | 17 (41.4) | 5 (35.7) | 12 (44.4) | 0.355 |
|  | Subthreshold | 13 (31.3) | 4 (28.5) | 9 (33.3) |  |
|  | Moderate | 9 (21.9) | 3 (21.4) | 6 (22.2) |  |
|  | Severe | 2 (4.8) | 2 (14.2) | 0 (0.0) |  |
|  |  |  |  |  |  |
| **Table S2b. Severity Categories of Depression, Anxiety, and Insomnia Measurements in Male PD Patients and Controls** | | | | | |
| **[Male]** | | **No. (%)** |  |  |  |
| **Severity category** | | **Total** | **PD** | **Control** | **P-value*** |
| PHQ-9, depression symptoms | | n = 29 | n = 24 | n = 5 |  |
|  | Normal | 12 (41.3) | 8 (33.3) | 4 (80.0) | 0.558 |
|  | Mild | 8 (27.5) | 8 (33.3) | 0 (0) |  |
|  | Moderate | 6 (20.6) | 6 (25.0) | 0 (0) |  |
|  | Severe | 3 (10.3) | 2 (8.3) | 1 (20.0) |  |
| GAD-7, anxiety symptoms | | n = 30 | n = 25 | n = 5 |  |
|  | Normal | 15 (50.0) | 11 (44.0) | 4 (80.0) | 0.249 |
|  | Mild | 7 (23.3) | 7 (28.0) | 0 (0) |  |
|  | Moderate | 6 (20.0) | 6 (24.0) | 0 (0) |  |
|  | Severe | 2 (6.6) | 1 (4.0) | 1 (20.0) |  |
| ISI, insomnia symptoms | | n = 30 | n = 25 | n = 5 |  |
|  | Absence | 14 (46.6) | 10 (40.0) | 4 (80.0) | 0.593 |
|  | Subthreshold | 7 (23.3) | 7 (28.0) | 0 (0) |  |
|  | Moderate | 9 (30.0) | 8 (32.0) | 1 (20.0) |  |
|  | Severe | 0 (0) | 0 (0) | 0 (0) |  |
| Abbreviations: PHQ-9, 9-item Patient Health Questionnaire; GAD-7, 7-item Generalized Anxiety Disorder; ISI, 7-item Insomnia Severity Index. | | | | | |
| * Cutoff scores for PHQ-9, GAD-7, and ISI were 10, 7, and 15, respectively. Participants who had scores greater than the cutoff threshold were characterized as showing clinical symptoms. Chi-squared test was applied to compare the rate of participants with clinical symptoms in PD patients and controls. | | | | | |
