# Supplementary material for: Risk factors for neuropsychiatric symptoms in patients with Parkinson’s disease during COVID-19 pandemic in Japan
Source: PLoS One. 2021 Jan 22;16(1):e0245864. doi: 10.1371/journal.pone.0245864 (PMC7822544; doi:10.1371/journal.pone.0245864)
Supplement: S3 Table — a. Scores of depression, anxiety, and insomnia measurements in female PD patients and controls. b. Scores of depression, anxiety, and insomnia measurements in male PD patients and controls. (DOCX) [file pone.0245864.s003.docx]

| **Table S3a. Scores of Depression, Anxiety, and Insomnia Measurements in Female PD Patients and Controls** | | | | |
| --- | --- | --- | --- | --- |
| **[Female]** | **Median (IQR)** | | |  |
| **Scale** | **Total score** | **PD** | **Control** | **P-value*** |
| PHQ-9, depression symptoms | 4.5 | 8.0 | 3.5 | 0.074 |
|  | (1.0-7.0) | (2.0-17.5) | (0.25-7.0) |  |
| GAD-7, anxiety symptoms | 5.0 | 6.0 | 5.0 | 0.28 |
|  | (1.0-8.0) | (2.0-14.0) | (1.0-7.0) |  |
| ISI, Insomnia symptoms | 8.0 | 10.5 | 8.0 | 0.322 |
|  | (3.0-15.0) | (3.5-17.5) | (2.5-13.5) |  |
|  |  |  |  |  |
| **Table S3b. Scores of Depression, Anxiety, and Insomnia Measurements in Male PD Patients and Controls** | | | | |
| **[Male]** | **Median (IQR)** | | |  |
| **Scale** | **Total score** | **PD** | **Control** | **P-value*** |
| PHQ-9, depression symptoms | 6.0 | 7.0 | 1.0 | 0.126 |
|  | (1.0-10.0) | (2.0-10.75) | (0.0-2.0) |  |
| GAD-7, anxiety symptoms | 5.0 | 6.0 | 2.0 | 0.557 |
|  | (1.0-9.75) | (1.0-10.0) | (1.0-4.0) |  |
| ISI, Insomnia symptoms | 8.5 | 10.0 | 2.0 | 0.277 |
|  | (2.75-15.0) | (5.0-15.0) | (2.0-6.0) |  |
| Abbreviations: PD, Parkinson's disease; IQR, interquartile range; PHQ-9, 9-item Patient Health Questionnaire; GAD-7, 7-item Generalized Anxiety Disorder; ISI, 7-item Insomnia Severity Index. | | | | |
| * Mann-Whitney U test was adapted to compare each questionnaire score between PD patients and controls on. | | | | |
