# Supplementary material for: Risk factors for neuropsychiatric symptoms in patients with Parkinson’s disease during COVID-19 pandemic in Japan
Source: PLoS One. 2021 Jan 22;16(1):e0245864. doi: 10.1371/journal.pone.0245864 (PMC7822544; doi:10.1371/journal.pone.0245864)
Supplement: S4 Table — a. Rate of female responders with worsening of motor performance, anxiety, and insomnia. b. Rate of male responders with worsening of motor performance, anxiety, and insomnia. (DOCX) [file pone.0245864.s004.docx]

| **Table S4a. Rate of Female Responders with Worsening of Motor Performance, Anxiety, and Insomnia** | | | | | |
| --- | --- | --- | --- | --- | --- |
| **[Female]** | | **No. (%)** |  |  |  |
| **Values** | | **Total** | **PD** | **Control** | **P-value** |
| Overall | | 41 (100) | 14 (34.1) | 27 (65.8) |  |
|  | Worsening of motor performance | 19 (46.3) | 8 (57.1) | 11 (40.7) | 0.370 |
|  | Worsening of anxiety | 19 (46.3) | 7 (50.0) | 12 (44.4) | 0.735 |
|  | Worsening of insomnia | 5 (12.1) | 3 (21.4) | 2 (7.4) | 0.193 |
| **Table S4b. Rate of Male Responders with Worsening of Motor Performance, Anxiety, and Insomnia** | | | | | |
| **[Male]** | | **No. (%)** |  |  |  |
| **Values** | | **Total** | **PD** | **Control** | **P-value** |
| Overall | | 30 (100) | 25 (54.9) | 5 (45.0) |  |
|  | Worsening of motor performance | 9 (30.0) | 8 (15.0) | 1 (20.0) | 0.593 |
|  | Worsening of anxiety | 7 (23.3) | 5 (20.0) | 2 (40.0) | 0.334 |
|  | Worsening of insomnia | 3 (10.0) | 2 (8.0) | 1 (20.0) | 0.414 |
| Abbreviation: PD, Parkinson's disease. | | | | | |
